# The HysNiche trial: hysteroscopic resection of uterine caesarean scar defect (niche) in patients with abnormal bleeding, a randomised controlled trial

**DOI:** 10.1186/s12905-015-0260-8

**Published:** 2015-11-12

**Authors:** A. J. M. W. Vervoort, L. F. Van der Voet, M. Witmer, A. L. Thurkow, C. M. Radder, P. J. M. van Kesteren, H. W. P. Quartero, W. K. H. Kuchenbecker, M. Y. Bongers, P. M. A. J. Geomini, L. H. M. de Vleeschouwer, M. H. A. van Hooff, H. A. A. M. van Vliet, S. Veersema, W. B. Renes, H. S. van Meurs, J. Bosmans, K. Oude Rengerink, H. A. M. Brölmann, B. W. J. Mol, J. A. F. Huirne

**Affiliations:** Department of Obstetrics and Gynaecology, VU medical centre, Amsterdam, Netherlands; Department of Obstetrics and Gynaecology, Deventer Hospital, Deventer, Netherlands; Department of Obstetrics and Gynaecology, Sint Lucas Andreas Hospital, Amsterdam, Netherlands; Department of Obstetrics and Gynaecology, Onze Lieve Vrouwe Gasthuis, Amsterdam, Netherlands; Department of Obstetrics and Gynaecology, Medical Spectrum Twente, Enschede, Netherlands; Department of Obstetrics and Gynaecology, Isala clinics, Zwolle, Netherlands; Department of Obstetrics and Gynaecology, Maxima Medical Centre, Eindhoven, Netherlands; Department of Obstetrics and Gynaecology, Sint Fransiscus Gasthuis, Rotterdam, Netherlands; Department of Obstetrics and Gynaecology, Catharina Hospital, Eindhoven, Netherlands; Department of Obstetrics and Gynaecology, Sint Antonius Hospital, Nieuwegein, Netherlands; Department of Obstetrics and Gynaecology, IJsselland hospital, Capelle a/d/ IJssel, Netherlands; Department of Obstetrics and Gynaecology, Academic Medical Centre, Amsterdam, Netherlands; Department of Health sciences and the EMGO Institute for Health and Care Research, VU University, Amsterdam, Netherlands; The Robinson Research Institute | School of Paediatrics and Reproductive Health University of Adelaide, Adelaide, Australia

**Keywords:** Niche, Caesarean section, Scar defect, Abnormal uterine bleeding, Postmenstrual spotting, Hysteroscopic resection

## Abstract

**Background:**

A caesarean section (CS) can cause a defect or disruption of the myometrium at the site of the uterine scar, called a niche. In recent years, an association between a niche and postmenstrual spotting after a CS has been demonstrated. Hysteroscopic resection of these niches is thought to reduce spotting and menstrual pain. However, there are no randomised trials assessing the effectiveness of a hysteroscopic niche resection.

**Methods/Design:**

We planned a multicentre randomised trial comparing hysteroscopic niche resection to no intervention. We study women with postmenstrual spotting after a CS and a niche with a residual myometrium of at least 3 mm during sonohysterography. After informed consent is obtained, eligible women will be randomly allocated to hysteroscopic resection of the niche or expectant management for 6 months.

The primary outcome is the number of days with postmenstrual spotting during one menstrual cycle 6 months after randomisation. Secondary outcomes are menstrual characteristics, menstruation related pain and experienced discomfort due to spotting or menstrual pain, quality of life, patient satisfaction, sexual function, urological symptoms, medical consultations, medication use, complications, lost productivity and medical costs. Measurements will be performed at baseline and at 3 and 6 months after randomisation. A cost-effectiveness analysis will be performed from a societal perspective at 6 months after randomisation.

**Discussion:**

This trial will provide insight in the (cost)effectiveness of hysteroscopic resection of a niche versus expectant management in women who have postmenstrual spotting and a niche with sufficient residual myometrium to perform a hysteroscopic niche resection.

**Trial registration:**

Dutch Trial Register NTR3269. Registered 1 February 2012. ZonMw Grant number 80-82305-97-12030

## Background

Caesarean section (CS) rates are rising globally. In the UK the CS rate increased from 12 to 29 % between 1990 and 2008 [1]. In the United States, one in three women delivered by CS in 2011 [1, 2], whereas in China the rates have even risen from 2 % in 1985 to 36–58 % in 2010 [3] The strongest increase was seen in Brazil from 15 % in 1970 to even 80 % in the private sector in 2004 [4]. This increasing CS rate has stimulated an interest in the potential long-term morbidity of CS scars.

**Fig. 1:**
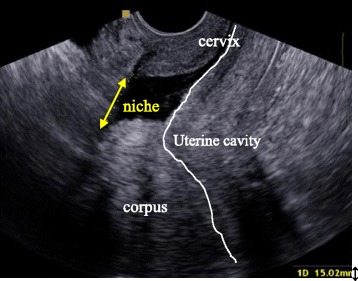
Sonohysterographic visualisation of a niche. The arrow indicates a disruption of the myometrium in the anterior wall of the uterus over a distance of approximately 15 mm

BijdeVaate et al. and van der Voet et al. reported a disruption of the myometrium in the uterine scar of a CS seen with saline or gel infusion sonography in approximately 60 % of women who had undergone a CS [5, 6]. Such a disruption or “defect” is called a niche (Fig. 1) [7]. Studies reported sonohysterography to be the most accurate method to identify and measure a niche [5, 6, 8]. Small niches, that are also reported to be associated with bleeding symptoms, can be missed using transvaginal sonography without contrast [5, 6].

Prolonged menstrual bleeding and postmenstrual spotting are associated with a niche [5, 6, 9–13]. Two independent cohort studies reported that approximately 30 % of women with a niche experience postmenstrual spotting of more than 2 days versus approximately 15 % of women without a niche after CS [5, 6].

**Fig. 2:**
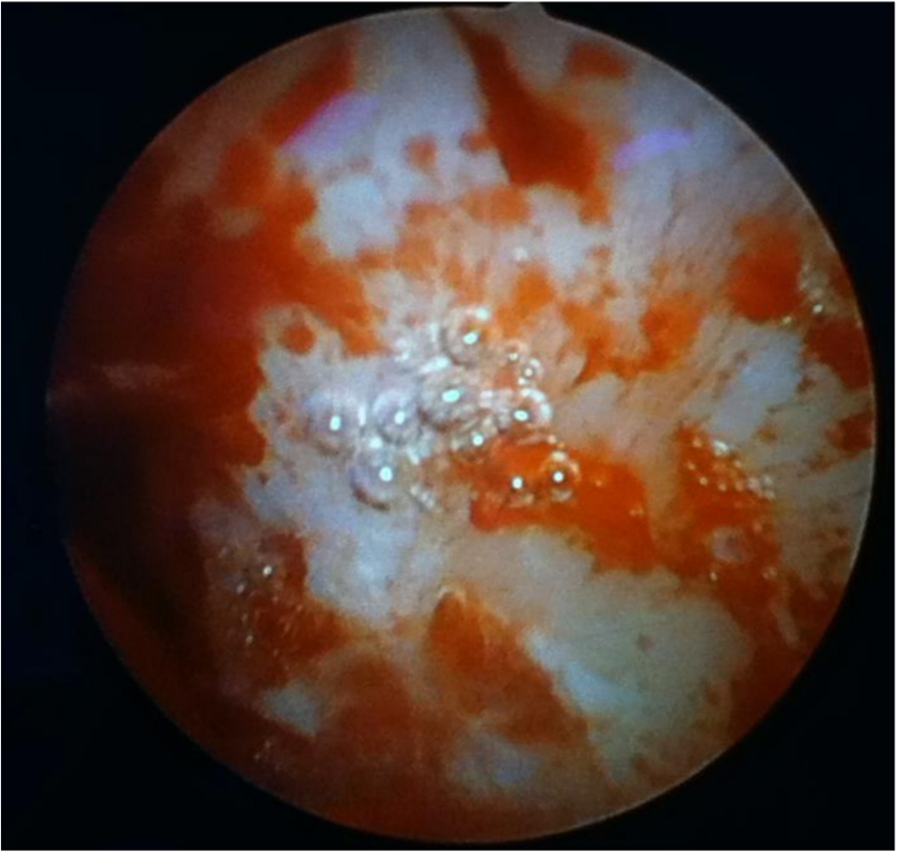
Niche surface during hysteroscopic evaluation of proximal part of the niche, several small vessels that easily bleed can be visualised

The etiology of niche related postmenstrual spotting and pain has not been fully elucidated. They are thought to be caused by retention of menstrual blood in a niche, which is intermittently expelled after the majority of the menstruation has ceased [11, 14–16]. Blood can also accumulate, if fibrotic tissue in the myometrium at the site of the caesarean scar may impair normal contractions and as a consequence the drainage of menstrual flow [11]. Additionally, newly formed fragile vessels in the niche may also attribute to the accumulation of blood or fluid in the niche or uterine cavity due to a constant low production of in situ leakage of blood and fluid. This is supported by the presence of free blood cells in the endometrial stroma, suggesting recent haemorrhage [17] and hysteroscopic evaluations where small vessels in the majority of patients are seen (Fig. 2) [15, 18–23].

A few studies reported that niche related menstrual bleeding disorders or cyclic pain do often not respond to hormonal therapies, however some comments can be made concerning the methodology of these studies [22, 24]. Some authors have recommended hysterectomy as a treatment for niche related bleeding complaints, but the exact number of patients undergoing a hysterectomy due to niche related bleeding disorders is yet unknown [11, 14, 25]. It is expected that niche related symptoms are associated to considerable direct and indirect costs, taking into account medical consultation, therapy (including hysterectomy) and absence from work.

Since many reports identified an association between niches and menstrual bleeding disorders after CS [5, 6, 9, 12, 13] several surgical therapies i.e. laparoscopic or hysteroscopic nicheresection or (laparoscopic assisted) vaginal niche repair, have been developed [26]. Of these treatments, hysteroscopic resection of the niche is the least invasive one. Resecting the distal rim aims at improving outflow of menstrual blood. Concurrently superficial coagulation of vessels in the niche aims at reducing blood loss from these fragile vessels.

So far only a few studies reported on a hysteroscopic resection of the niche with coagulation of the niche surface [16, 17, 20–24]. These procedures were reported to improve abnormal blood loss in 87 %, improve pain complaints in 97 % of the cases and no complications occurred [26]. The reduction in spotting days after the procedure compared to baseline was reported in three studies, mean reduction in spotting varied from 2 to 4 days [18, 21, 27].

Thus reported outcomes are promising. However, the methodological quality of these studies is low. Follow-up or validated tools to measure outcomes such as blood loss or quality of life are mostly not reported, most studies are case series or retrospective cohorts and adequately powered prospective comparative studies are lacking [26]. In addition, none of the studies evaluated the effect of hysteroscopic niche resection on quality of life, sexual function, recovery or cost. Before hysteroscopic niche resection is implemented in women that suffer from postmenstrual spotting based on a niche, better assessment of the effectiveness of the treatment is needed.

In view of these arguments, we designed a randomised controlled trial to evaluate the (cost) effectiveness of a hysteroscopic niche resection.

## Methods/Design

### Design

The study is a multicentre randomised controlled superiority trial and will be performed in hospitals that collaborate within the Dutch Consortium for Studies in Women’s Health and Reproduction. Participating centres can be district, teaching or university hospitals. The hospitals are selected based on the presence of extensive experience of their gynaecologists in advanced hysteroscopic procedures, such as hysteroscopic resections of large submucosal fibroids. Gynaecologists will be additionally trained in their centre in the measurement of a niche and in performing a hysteroscopic niche resection by one of the experienced gynaecologists that performed niche resections in a previous pilot study (J. Huirne, L. van der Voet or H. Brölmann).

One of the experienced gynaecologists will be present during the first hysteroscopic niche resection and if needed also during following hysteroscopic niche resections in each centre.

### Participants/eligibility criteria

We will study women who developed postmenstrual spotting after a CS, and in whom sonohysterography has shown a niche of at least 2 mm depth and a residual myometrium of at least 3 mm. Postmenstrual spotting is defined as two or more days of intermenstrual spotting or as two or more days of brownish discharge immediately after the menstrual period in case the total period of the menstrual bleeding exceeds 7 days, if the period is shorter then 7 days the brownish discharge is considered to be normal.

In these women, saline or gel sonohysterography (SIS or GIS) will be performed as part of routine diagnostic work-up for identification of the origin of the bleeding. Exclusion criteria are a residual myometrium less than 3 mm at sonohysterography, age below 18 years, pregnancy, a (suspected) malignancy, contra-indications for spinal of general anaesthesia, uterine or cervical polyps, submucosal fibroids, atypical endometrial cells, cervical dysplasia, cervical or pelvic infection, hydrosalpinx that communicates with the uterus or an irregular cycle (>35 days or intercycle variation of 2 weeks or more).

### Procedures, recruitment, randomisation, collection of baseline data

**Fig. 3:**
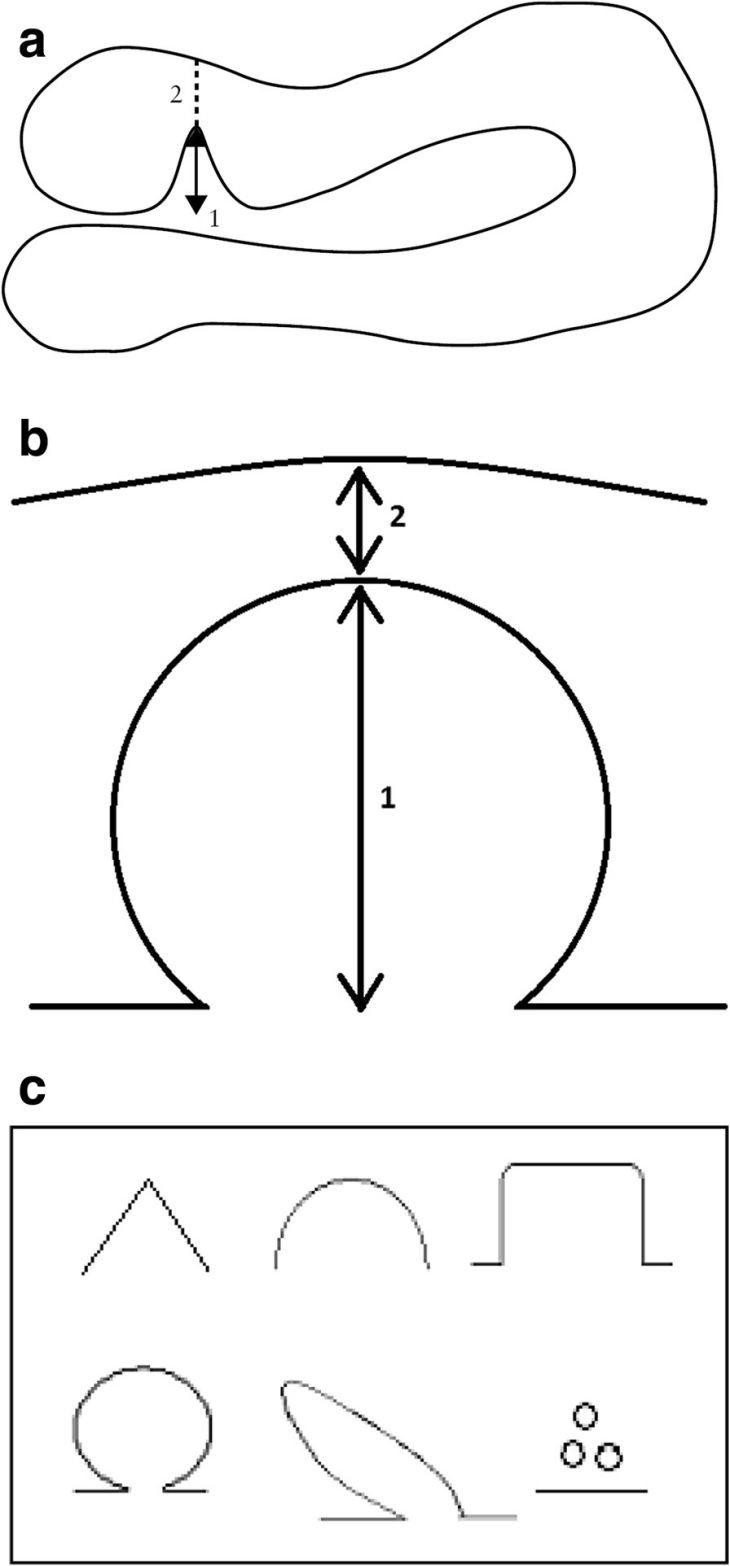
Niche measurement during sonohysterography in the sagittal plane (**a**), transversal plane (**b**) with the thinnest residual myometrium and niche shape will be registered in both planes (**c**). **a** Measuring a niche in the sagittal plane. Schematic drawing demonstrating how to measure a niche in the sagittal plane. The depth of the niche is measured from the usual limit of the uterine cavity until the apex of the niche (1), the residual myometrium from the apex of the niche until the serosa (2). **a** is an adapted figure of the one that was published by Bij de Vaate et al. 2011 [5]. **b** Measuring a niche in the transversal plane. Schematic drawing demonstrating how to measure a niche in the transversal plane. The depth of the niche is measured from the usual limit of the uterine cavity until the apex of the niche (1) and the residual myometrium from the apex of the niche until the serosa (2). **c** Niche shape. Schematic diagram demonstrating classification used to assess niche shape as published by Bij de Vaate et al. 2011: triangle, semicircle, rectangle, circle, droplet and inclusion cysts [5]

Before study entry, the niche will be measured with transvaginal sonography and saline or gel infusion sonography in the sagittal plane where the largest niche is visible and in the transversal plane where the niche is largest i.e. with the thinnest residual myometrium. Depth, residual myometrium and the shape of the niche will be registered (see Fig. 3a, b and c). For inclusion, a niche needs to be at least 2 mm deep and the residual myometrium should not be less than 3 mm in one of the planes during SIS or GIS, given the anticipated risk on perforation or bladder injuries with a smaller residual myometrium. Position of the uterus, fluid in the uterine cavity and/or niche, abnormalities of the fallopian tubes and/or ovaries are also registered. A cervical PAP-smear will be obtained to ensure the absence of cervical dysplasia.

Eligible women will be informed about the study by one of the gynaecologists, residents or research nurse in the participating centre. The informed consent form must be signed before involvement in any study related activity. After written informed consent is obtained, eligible women will be randomly allocated to hysteroscopic resection (experimental intervention) or no intervention (control). Randomisation will be performed by using ALEA, an online randomisation program, managed by the Clinical Research Unit in the Academic Medical Centre in Amsterdam, with the use of permuted blocks with a random block size of 2 to 4 patients per block, stratified for participating centres.

At the time of randomisation, baseline, obstetric and medical history are reported in the case report file. The patient will receive digital online secured questionnaires at baseline, 3 and 6 months after randomisation about their menstrual pattern and uterine bleeding, will complete a validated menstrual bleeding score chart [28], validated questions to report on quality of life (SF-36: Short Form 36 [29, 30] and Euro-QOL 5D: Euro Quality of Life in 5 dimensions [31]), sexual function (FSFI: Female Sexual Function Index) [32] and absence from work (HLQ: Health and Labour [33]). Medical consultation, medication use and received additional therapies will be registered at the same timepoints in an online diary. Any received additional therapy will be registered in the case report file.

**Fig. 4:**
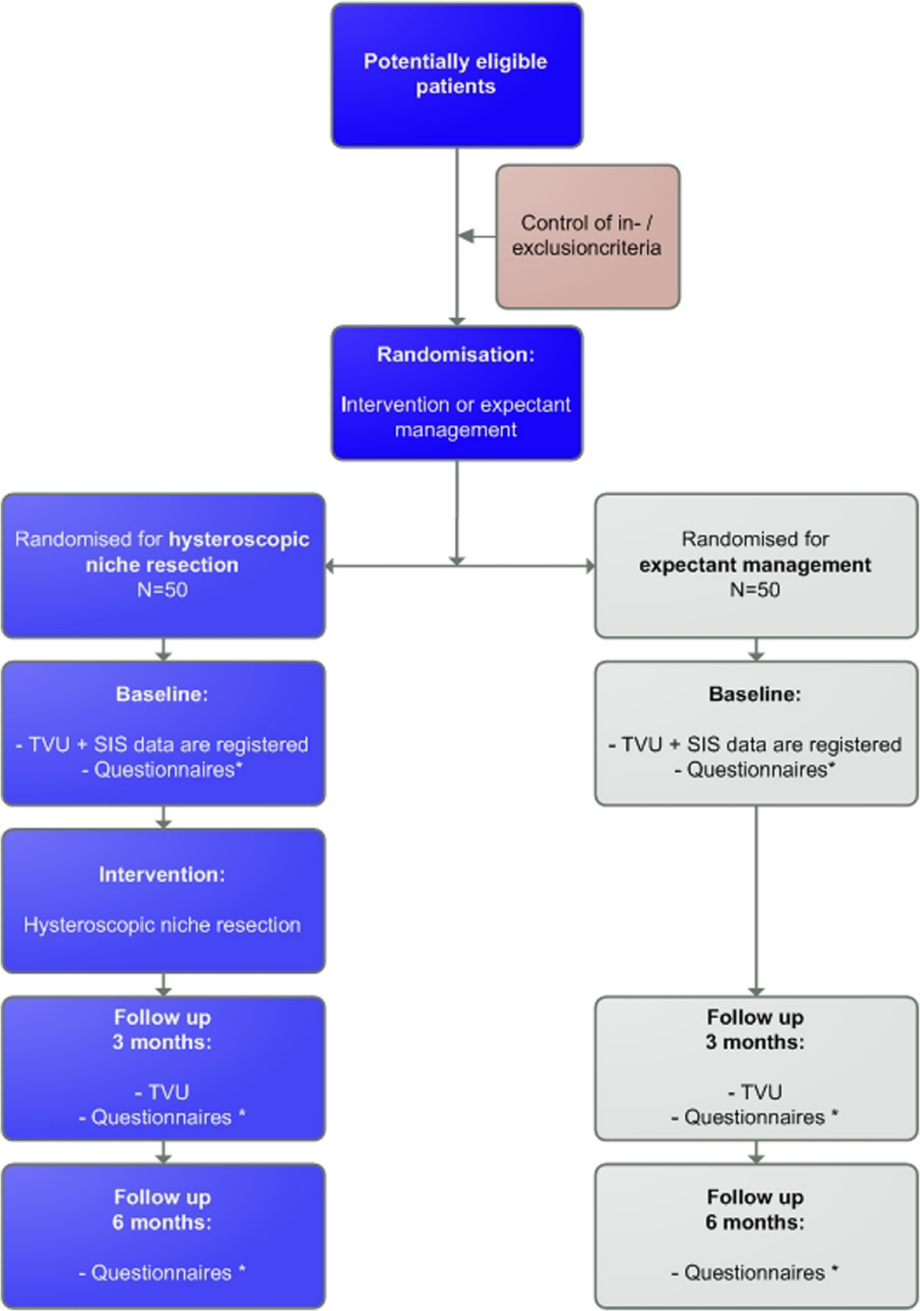
Flowchart. * Questionnaires: Menstruation questionnaire, SF-36 (Short Form-36 [29, 30]), Euro-Qol 5D [31], FSFI (Female Sexual Function Index) [32], menstruation chart [28], costs and consultation diary, HLQ (Health and Labour Questionnaire) [33]

Three months after randomisation all patients will undergo transvaginal sonography to register all niche features as at baseline. Patients will be motivated to undergo a SIS or GIS, however saline or gel instillation will be omitted in case patients experienced too much discomfort during this procedure at baseline (see Fig. 4 for the flowchart).

### Intervention (hysteroscopic niche resection)

**Fig. 5:**
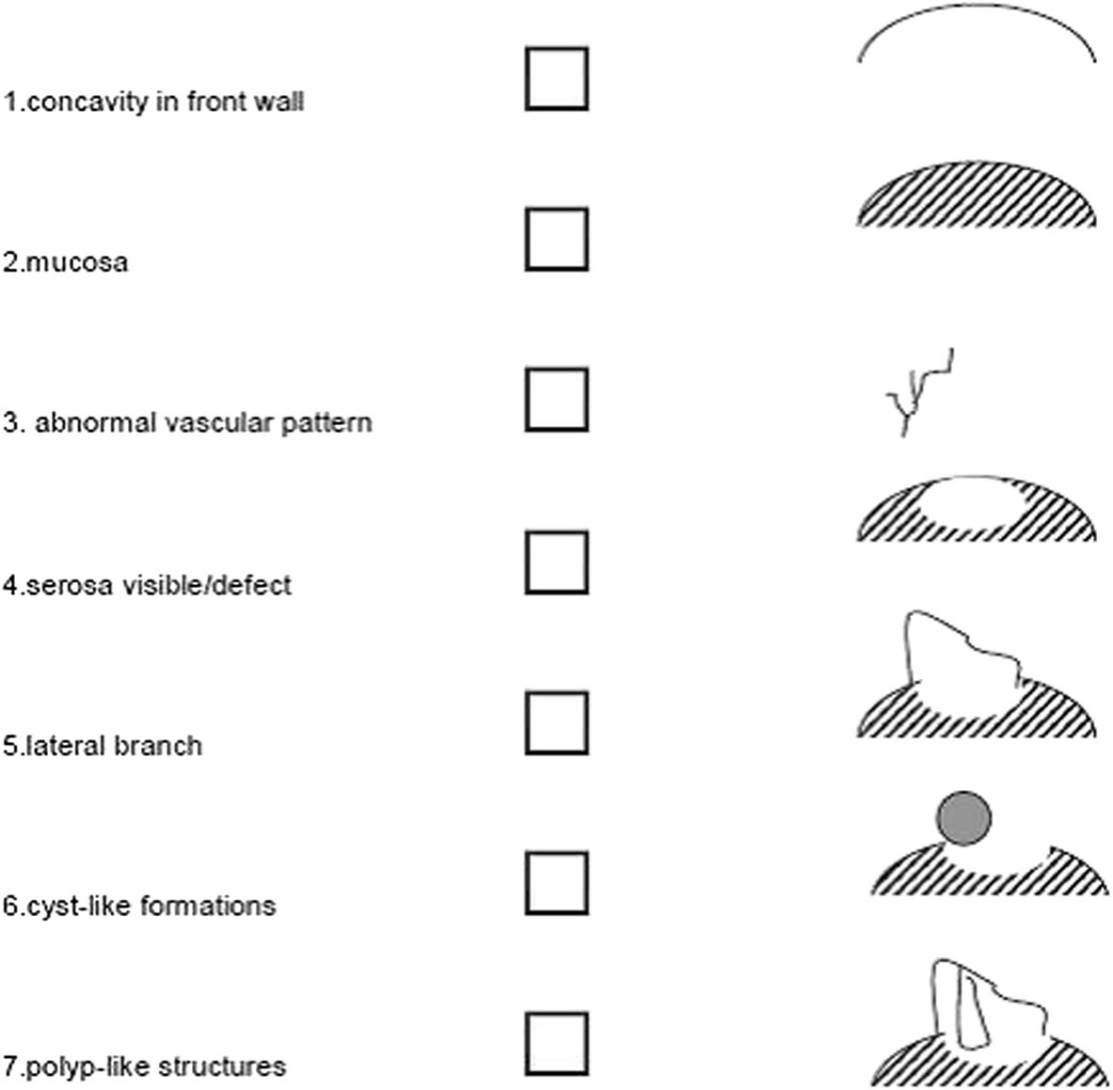
Schematic diagram of niche characteristics during hysteroscopy

The patients allocated for hysteroscopic niche resection will undergo the procedure under spinal or general anaesthesia in ambulatory setting. After dilatation of the cervical os up to Hegar 9 mm, the hysteroscopic resection will be performed using a 9 mm resectoscope in a standardized way, as described below. In case of bipolar currency, 0.9 % NaCL and in case of monopolar currency, sorbitol fluid will be used to induce distension of the uterine cavity. The niche will be evaluated by hysteroscopy and standardized characteristics will be registered (Fig. 5).

**Fig. 6:**
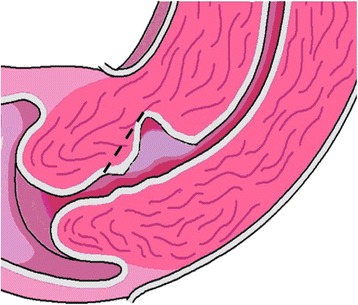
Resection of the distal part of the niche. Is a modified figure of the one that was published by Gubbini et al. 2008 [18] and v.d. Voet et al. [26]

The bladder will be filled with methylene blue solution during the procedure to enable early identification of eventual bladder injuries. The procedure will be performed under continuous combined sonographic evaluation to ensure sufficient distance between the resection and coagulation area and the bladder. The distal rim of the niche in utero, if prominent visible, will be resected (Fig. 6) as described by i.a. Chang et al. and Fabres et al. and the niche surface will be superficially coagulated. Eventual polyps in the niche will be resected. A maximum fluid loss of 1000 ml Sorbitol or 2000 ml NaCl is accepted. Surgical outcomes such as satisfaction of the gynaecologist, surgical steps, complications and postoperative hospital stay will be registered in the case report file. Name and experience in numbers of resections of the gynaecologist with hysteroscopic niche resections will be registered. All gynaecologists will be evaluated in performing the resection using an Objective Structured Assessment of Technical Skills (OSATS). In case of an uneventful procedure, patients will be discharged on the same day.

### Control group (expectant management)

The control group will receive no additional intervention during the first six months after randomisation. Patients will be encouraged to continue the medication and/or oral contraceptives during this period as used before randomisation. Six months after randomisation all types of additional therapies are allowed. All additional therapies given up to six months will be registered in both the intervention and the control group, such as oral contraceptives, IUD placement or hysterectomy.

### Outcome measures

The primary outcome is the number of days with postmenstrual spotting during one menstrual cycle 6 months after randomisation.

Secondary outcomes are the menstrual characteristics, menstrual related pain and experienced discomfort of both spotting and menstrual pain on VAS of 0–10), quality of life (SF-36, EuroQoL5D, patient satisfaction (5 point scale), sexual function (FSFI), micturition frequency, and urinary incontinence, absence from work (HLQ: Health and Labour [33]), medical consultation, medication use and received additional therapies (diary) at 3 and 6 months after randomisation.

#### Sample size

Based on a systematic review of the literature executed in 2011 we identified one relevant manuscript reporting on a reduction in postmenstrual abnormal uterine bloodloss (PAUB) with an estimated median reduction of 3.8 days after a hysteroscopic niche resection compared to baseline [27]. At baseline, included patients had mean spotting of 7.5 days with a standard deviation of 2.7. Estimated postmenstrual spotting reduced from 7.5 to 3.7 days (see box and whisker plots). Given the fact that the ranges at baseline and after therapy were more or less similar we estimated the SD to be approximately 2.7. However given the lack of a control group without treatment it cannot be excluded that regression to the mean have played a role and may have induced an overestimation of the effect. Given these uncertainties we decided to base our sample size on the difference in postmenstrual spotting days that were considered to be clinically relevant and this should be lower than the previously reported difference of 3.8 in order to prevent insufficient power. Based on experience and confirmed in a survey among patients (unpublished), we considered 2 days to be clinically relevant and a dropout rate of 20 % to be realistic and used these parameters to calculate a sample size to achieve a power of 90 %.

In summary, assuming a difference of postmenstrual spotting of 2 days to be clinical relevant with an estimated SD of 2.7, a dropout rate of 20 %, we need to include 50 patients in each arm to achieve a power of 90 %.

#### Statistical analysis

Analyses will be performed according to intention-to-treat principle and additionally by per protocol analysis. All tests will be performed two sided and a *p*-value <0.05 will be considered as statistically significant. The difference between the two groups at 6 months follow-up regarding total number of postmenstrual spotting days during one menstrual cycle, duration of menstrual bleeding, number of days of brownish discharge, menstrual pain, experienced discomfort of spotting and of menstrual pain, SF-36 domain scores and EuroQol will be analysed using linear regression analyses, adjusted for relevant baseline values. Potentially relevant confounders are additional hormonal therapy, smoking and use of anticoagulation. Logistic regression analysis will be used to evaluate the existence of (midcycle) intra-uterine fluid. To compare satisfaction with treatment, reported on a 0–5 point scale at six months a (multinominal) logistic regression analysis will be used.

We have planned four subgroup analyses, for:

1) Total days of spotting at the end of the menstruation and intermenstrual spotting >25^th^ centile versus ≤ 25^th^ centile and >75^th^ centile or ≤75^th^ centile at baseline;

2) Number of days postmenstrual spotting >25^th^ centile versus ≤2 25^th^ centile and >75^th^ centile versus ≤75^th^ centile at baseline;

3) Small versus larger niches (using a cut-off of residual myometrium of 3 and 5 mm and <50 % of total myometrial thickness (sum of RM and niche depth) at baseline;

4) A history of 1 versus more than 1 CS at baseline.

### Economic evaluation

The economic evaluation will be performed alongside the randomised controlled trial. Both a cost-effectiveness analysis as a cost-utility analysis will be performed from the societal perspective. In the cost-effectiveness analysis, the reduction in postmenstrual spotting days will be used as the primary outcome. In the cost-utility analysis, societal costs will be related to QALYs over 6 months. Costs that will be included are direct healthcare costs (costs of the hysteroscopic niche resection, use of healthcare services such as outpatient visits, hospital admissions, laboratory and imaging tests, visits to health care providers outside the hospital, medication, etcetera), direct non-healthcare costs (i.e. informal care), and indirect non-healthcare costs (absenteeism and presenteeism from paid and unpaid work).

We hypothesize that hysteroscopic niche resection will reduce postmenstrual spotting and related medical consultations, applied therapies and sick leave and as a consequence will be cost-effective in comparison with the expectant management strategy.

Analyses will be performed according to the intention-to-treat principle. Missing cost and effect data will be imputed using multiple imputation [34]. The imputation will include variables that are related to missing data or the outcome measure, and variables that differ at baseline between the groups. To account for the skewed distribution of costs predictive mean matching will be used in the multiple imputation. The number of imputed data sets to be created will be determined based on the fraction of missing information [35]. All datasets will be analyzed separately and the results of the 20 analyses will be pooled using Rubin’s rules [36]. Incremental cost effectiveness ratios (ICERs) will be calculated by dividing the differences in mean total costs between both treatment groups, by the difference in mean effects between both treatment groups [36]. To avoid double counting, productivity costs due to sick leave will be excluded in the ICER with sick leave as effect measure. The incremental cost utility ratio will be calculated by dividing the incremental costs by the difference in QALYs between both treatment groups. To account for the typically skewed distribution of costs, bias-corrected and accelerated bootstrapping (5000 replications) will be used to estimate the 95 % confidence intervals around the mean cost differences and the uncertainty surrounding the ICERs. The bootstrapped ICERs will be graphically presented in cost-effectiveness planes [37]. Cost-effectiveness acceptability curves will be estimated to show the probability of the intervention program to be cost-effective in comparison with usual care for a range of different ceiling ratios, thereby showing decision uncertainty [38].

### Interim analysis and safety monitoring

Because of the relatively small sample size and the expected duration of inclusion no interim analysis for efficacy is planned. All Serious Adverse Events (SAE’s) will be reported to the Medical Ethics Committee. In case of two Serious Adverse Events (SAE’s) related to the study intervention the Data Safety Monitoring Committee, will be notified. The DMC can advise to terminate the trial prematurely for safety reasons. All adverse events will be followed until they have abated, or until a stable situation has been reached. Depending on the event, follow up may require additional tests or medical procedures as indicated, and/or referral to the general physician or a medical specialist.

### Ethical considerations

This study is approved by the National Central Committee on Research involving Human Subjects (CCMO – NL38397.029.11), by the ethics committee of the VU Medical Centre Amsterdam (Ref. No. 2011/397) and by the boards of all participating hospitals. The trial is registered in the Dutch Trial Register (NTR3269, http://www.trialregister.nl).

## Discussion

Only recently, we became aware of an association between niches in the CS uterine scar and bleeding disorders [5, 6, 9–12]. Postmenstrual spotting is present in approximately 20 % of women after a CS. Several innovative surgical therapies to reduce these complaints have been developed [26]. Currently, the least invasive surgical therapy is the hysteroscopic resection of the niche. So far only a few studies reported on hysteroscopic resections. Success rates on reduction of postmenstrual spotting are high and no complications are reported [15, 18–23, 26, 27]. However exact methodology, follow-up or (validated) tools to measure outcomes are mostly not reported and sample sizes were small.

### Strengths and limitations

This trial is the first randomised controlled trial that will provide evidence for the (cost) effectiveness of hysteroscopic resection of a niche versus expectant management in women with niche related postmenstrual spotting.

The HysNiche study is adequately powered. Randomisation is performed with the use of allocation concealment through a webbased randomisation program, which reduces the chance for bias. The study is not blinded for the patient, which could possibly effect reported outcomes by the patient. We found it ethically not justified to ask patients to undergo a hysteroscopic procedure without performing the resection, because of the risk on complications due to the hysteroscopy or anesthesia and possible pain complaints after the procedure.

Furthermore the HysNiche study will evaluate quality of life and sexual function in relation to complaints of spotting, which was not reported in earlier studies.

None of earlier studies reported on cost-effectiveness of a hysteroscopic niche resection compared to expectant management. We assume a hysteroscopic niche resection to be more cost-effective, since we expect it to prevent the need for invasive surgical interventions such as a hysterectomy due to therapy resistant spotting or menstrual pain. We expect postoperative recovery time to be short and medical consultation to be less frequent. And not irrelevant, we think quality of life could improve with the knowledge of having low health care costs.

Questionnaires used are not adjusted or validated for spotting complaints. Disease specific validated questionnaires have not been developed yet.

Different methods of performing a hysteroscopic resection are described. In some studies only the distal rim of the niche is resected [15, 20, 21, 27], whereas in other studies the distal and proximal part of the niche is resected [18, 19, 22, 23]. In some studies the fragile vessels in the bottom of the niche are coagulated [18, 19, 21–23], while in other studies the entire niche surface is coagulated [15, 20]. It is unclear if authors used different descriptions of the same procedure (i.e. if the resection of the proximal part of the niche is the same as coagulation of the vessels in the entire niche surface. Given the fact that it can not be excluded that proximal resection could harm the strength of the cervix and may induce unneeded cervical incompetence in case of a subsequent pregnancy we decided to omit this part of the procedure. In addition niche resection is expected to increase the size of the niche and resection of both distal and proximal part may induce unneeded increase of the size of the niche. Based on theory we hypothesized that in particular the distal rim may impair normal outflow of menstrual blood. And as a consequence we expect that resection of the distal rim of the niche will improve blood flow towards the cervix. In addition we expect that superficial coagulation of the vessels of the niche surface is sufficient to prevent bleeding from potentially fragile niche vessels.

### Potential impact and implications

This trial will provide evidence for patients, healthcare providers and policy makers on the (cost) effectiveness of hysteroscopic resection of a niche versus expectant management in women with postmenstrual spotting and a niche with sufficient residual myometrium to perform a hysteroscopic niche resection. It is important to realize that not all niches cause symptoms, and as the treatment is predominantly performed to relieve symptoms, niches without symptoms should not be treated by a hysteroscopic niche resection. In addition it is important to realize that we only include patients with relatively small niches thus the outcomes can’t be extrapolated to patients with large niches. In patients with large niches, i.e. thin residual myometrium of less then 3 mm a hysteroscopic niche resection is expected to be at higher risk of bladder injury and uterine perforations.

Based on results of this study, implementation of the hysteroscopic niche resection in daily practice can be discussed and long-term complications of a CS can possibly be reduced. The patient reported outcomes could inform future patients about the expected outcomes of both niche resection and expectant management and support shared decision making.

## Abbreviations

CS: Caesarean section
SIS: Saline infused sonography
GIS: Gel infused sonography
SF-36: Short Form 36 (questionnaire for health status)
Euro-QOL: Euro-QOL (questionnaire for health status)
FSFI: Female Sexual Function Index
HLQ: Health and Labour questionnaire
DSMC: Data safety monitoring committee
SAE’s: Serious adverse events
